# Deep Learning-Based Classification of Common Lung Sounds via Auto-Detected Respiratory Cycles

**DOI:** 10.3390/bioengineering13020170

**Published:** 2026-01-30

**Authors:** Mustafa Alptekin Engin, Rukiye Uzun Arslan, İrem Senyer Yapici, Selim Aras, Ali Gangal

**Affiliations:** 1 Department of Electrical and Electronics Engineering, Bayburt University, 69000 Bayburt, Türkiye; maengin@bayburt.edu.tr; 2 Department of Electrical and Electronics Engineering, Zonguldak Bülent Ecevit University, 67100 Zonguldak, Türkiye; 3 Department of Computer Engineering, Zonguldak Bülent Ecevit University, 67100 Zonguldak, Türkiye; senyerirem@beun.edu.tr; 4 Department of Electrical and Electronics Engineering, Ondokuz Mayıs University, 55200 Samsun, Türkiye; selim.aras@omu.edu.tr; 5 Department of Electrical and Electronics Engineering, Karadeniz Technical University, 61080 Trabzon, Türkiye; ali.gangal@ktu.edu.tr

**Keywords:** lung sounds, respiratory cycle, automatic recognition, deep learning, time-frequency representations, classification

## Abstract

Chronic respiratory diseases, the third leading cause of mortality on a global scale, can be diagnosed at an early stage through non-invasive auscultation. However, effective manual differentiation of lung sounds (LSs) requires not only sharp auditory skills but also significant clinical experience. With technological advancements, artificial intelligence (AI) has demonstrated the capability to distinguish LSs with accuracy comparable to or surpassing that of human experts. This study broadly compares the methods used in AI-based LSs classification. Firstly, respiratory cycles—consisting of inhalation and exhalation parts in LSs of different lengths depending on individual variability, obtained and labelled under expert guidance—were automatically detected using a series of signal processing procedures and a database was obtained in this way. This database of common LSs was then classified using various time-frequency representations such as spectrograms, scalograms, Mel-spectrograms and gammatonegrams for comparison. The utilisation of proven, convolutional neural network (CNN)-based pre-trained models through the application of transfer learning facilitated the comparison, thereby enabling the acquisition of the features to be employed in the classification process. The performances of CNN, CNN and Long Short-Term Memory (LSTM) hybrid architecture and support vector machine methods were compared in the classification process. When the spectral structure of gammatonegrams, which capture the spectral structure of signals in the low-frequency range with high fidelity and their noise-resistant structures, is combined with a CNN architecture, the best classification accuracy of 97.3% ± 1.9 is obtained.

## 1. Introduction

The human respiratory system plays a pivotal role in human physiology, facilitating the transportation of oxygen to bodily cells and the elimination of carbon dioxide. Lung sounds (LSs), which are produced by the movement of air through the airways and lung tissue during respiration, are of considerable importance in the evaluation of respiratory system health. The advent of the stethoscope, pioneered by Laennec in 1816 [1], signified a pivotal advancement in the diagnostic evaluation of respiratory ailments through the auditory perception of lung sounds, thus establishing the stethoscope as a quintessential instrument in the diagnostic armamentarium of physicians [2].

In the diagnosis of respiratory diseases, common lung sounds are generally classified into five main types: normal, rales, fine crackles, coarse crackles and wheezes [3]. Normal breath sounds, which indicate airflow through the structural components of the lungs, are characterised by being soft and low-frequency and can be heard in different regions of the lungs. Rales, characterised by low-pitched, whistling or noisy sounds, are usually associated with airway narrowing. Fine crackles, associated with conditions such as pneumonia and pulmonary fibrosis, are high-frequency wheezing sounds caused by the sudden opening of small airways. Coarse crackles, on the other hand, are low-pitched, bubble-like sounds that usually occur in bronchitis and chronic obstructive pulmonary disease (COPD) due to significant mucus accumulation. Wheezes—high-pitched sounds typically heard during expiration—arise from airway narrowing or obstruction, often indicative of asthma or COPD. Given the diagnostic value of these distinct LS types, their analysis is vital for the early diagnosis and effective management of respiratory diseases. However, the delayed onset of noticeable symptoms in patients frequently results in diagnostic and treatment delays. Therefore, there is an increasing demand for faster and more efficient diagnostic methodologies. The stethoscope, used for over two centuries, remains essential for diagnosing lung disorders through auditory examination. Though the stethoscope aids in more accurate LS diagnosis, its effectiveness largely depends on the clinician’s expertise, introducing variability and potential errors, especially among less experienced practitioners. Indeed, Mangione and Nieman [4] highlighted that approximately fifty percent of LSs were misidentified by hospital trainees, with similar inaccuracies also observed among medical students. A follow-up study assessed the accuracy of respiratory sound categorisation among medical students, interns, residents and fellows. The study revealed mean correct response rates of 73.5% for normal sounds, 72.2% for crackles, 56.3% for wheezes and 41.7% for rhonchi, with rhonchi proving to be the most challenging sound to identify [5]. Additionally, the use of the stethoscope involves various limitations such as difficulties in detecting low-frequency sounds and the effects of environmental noise [6]. These limitations constitute the primary challenges traditionally encountered in the diagnosis of respiratory diseases. To address these shortcomings, digital stethoscopes are being utilised to record LS and employ artificial intelligence (AI)-assisted analysis methods with signal processing techniques. These methods can serve as a decision support tool, providing a second opinion that helps validate clinical judgements and significantly reduces the risk of human error [7,8]. Therefore, a review of existing studies reveals that various artificial intelligence techniques, such as deep learning (DL), are being used for the automatic identification and classification of respiratory sounds. By using Mel-spectrograms to detect respiratory anomalies, Acharya and Basu [9] proposed a Convolutional Neural Network−Recurrent Neural Network (CNN-RNN) model. Thanks to patient-specific adjustments, the model’s accuracy increased from 66.31% to 71.81%. Nguyen and Pernkopf [10] presented an efficient system using a set of snapshots from CNNs trained on log Mel-spectrograms to recognise LSs. The system utilised a cosine cycle learning rate and applied data augmentation with focal loss (FL) to address class imbalance. It achieved micro-averaged accuracies of 83.7% for two-class tasks and 78.4% for four-class tasks. Er [11] introduced a CNN-based method for LS classification, achieving a 64.5% accuracy. Spectrograms of pre-processed audio signals were fed into a 12-layer CNN for feature extraction. Demir et al. [12] proposed a hybrid approach that used a pre-trained CNN for deep feature extraction, incorporating parallel average and max-pooling layers. The extracted features were classified via Linear Discriminant Analysis (LDA) combined with Random Subspace Ensembles, achieving a 5.75% improvement in accuracy over existing methods. Jung et al. [13] introduced a feature engineering strategy to optimise a depthwise separable convolutional neural network (DS-CNN) for the analysis of LSs. The study utilised three feature sets: short-time Fourier transform (STFT), MFCC and a fused combination of both. The DS-CNN trained on the fused features achieved an accuracy of 85.74%, surpassing models based on individual features. Kim et al. [5] developed a CNN-based model for classifying respiratory sounds, recorded in clinical settings. Using transfer learning (TL) with pre-trained image features, the model achieved an 86.5% accuracy in detecting abnormal sounds and an 85.7% accuracy in classifying them, highlighting its potential to enhance clinical auscultation and aid in diagnosing respiratory diseases. Lang et al. [14] introduced Graph Semi-Supervised CNNs (GS-CNNs) to classify LSs using a small labelled dataset combined with a larger unlabelled set. A graph of respiratory sounds was constructed to capture relationships among samples, and its information was integrated into the loss function of a four-layer CNN. Gupta et al. [15] developed a new preprocessing technique to remove respiratory noise using variational mode decomposition. The signals were converted into gammatonegram images with a Gammatone filter bank for time-frequency analysis. Various CNN architectures were employed for classification via TL to address the challenge of limited dataset sizes. The proposed method achieved an impressive accuracy of 98.8%. Tariq et al. [16] proposed a feature-based fusion network (FDC-FS) for LS classification, leveraging TL from three deep neural networks (DNNs). Audio data were transformed into image vectors using spectrogram, MFCC and chromagram features, which were combined into a fusion model that achieved a 99.1% accuracy. Petmezas et al. [17] developed a hybrid neural network model incorporating a fully connected layer to address data imbalance in LS classification. STFT spectrogram features were processed via a convolutional neural network and then fed into a Long Short-Term Memory (LSTM) network to capture temporal dependencies, achieving a classification accuracy of 76.39%. Pham Thi Viet et al. [18] employed a scalogram-based CNN approach for LS classification, introduced a novel inverse sample filling method and applied data augmentation directly to scalograms. Engin et al. [19] proposed a fully automatic method for classifying single-channel LSs using automatic detection of respiratory cycles. The study combined MFCC, linear predictive coding (LPC) and time-frequency domain features and was optimised through sequential forward selection. Using a combination of LPC and MFCC features with a k-NN classifier, it achieved the highest accuracy of 90.14% in training and 90.63% in testing. Yang et al. [20] presented a new DNN model (Blnet) that integrates ResNet, GoogleNet and a self-attention mechanism to improve respiratory sound classification. Blnet achieved an overall performance score of 72.72%, representing a 4.22% improvement over the compared methods. Cinyol et al. [21] analysed the integration of SVM into CNN for multi-class respiratory sound classification. They achieved an 83% classification accuracy using 10-fold cross-validation and VGG16-CNN-SVM. Khan et al. [22] used continuous wavelet transform (CWT) and Mel-spectrograms to classify respiratory diseases using LSs, and created scalograms processed by parallel convolutional autoencoders. The features obtained were then classified using an LSTM network. The proposed model achieved high accuracy in eight-class (94.16%), four-class (79.61%) and binary-class (85.61%) classification processes. Wu et al. [23] introduced a Bi-ResNet DL model that integrates CNNs and Residual Networks (ResNets) using STFT and WT during the feature extraction stage to classify LSs. Achieving a classification accuracy of 77.81%, it recorded a 25.02% improvement over the standard Bi-ResNet model. Zhang and Liu [24] proposed a CNN-CatBoost model that utilises pre-trained VGG19 parameters for LS recognition, reducing overfitting with limited data and employing the Convolutional Block Attention Module (CBAM) for enhanced performance, achieving a classification accuracy of 75.73%.

In the studies aforementioned, LSs were collected under different techniques and conditions, focusing on specific age groups and a limited number of patients. These limited conditions also apply to commonly used datasets such as the ICBHI [25] and RALE [26] databases, which are restricted in terms of abnormal sound types. For example, while the ICBHI database is limited to rales and wheezes recordings, the RALE database does not include rhonchi sounds [5]. In particular, wheeze sounds can be detected without the use of a stethoscope due to their distinctive features, which are much more dominant than other common lung sounds, so including a large number of samples of this sound group in the database will lead to biased average results in classification success. Another limitation of the studies is the imbalance in the number of samples belonging to each class. Furthermore, the segmentation of respiratory sounds based on predefined durations introduces an additional constraint, as the varying duration of respiration across individuals has the potential to inadvertently divide respiratory cycles, resulting in inconsistencies. Conversely, single-channel and multichannel methods employed in the recording of respiratory sounds possess distinct advantages and disadvantages when compared to each other. While multichannel systems facilitate the acquisition of a more substantial dataset, they present challenges in terms of the placement of receivers on the patient’s body, particularly in cases where the patient is overweight or possesses a high hair density. Conversely, single-channel systems, despite their ease of application, are constrained by limitations in the amount of data that can be obtained. In order to surmount the aforementioned difficulties, this study employed a standardised methodology in which breathing cycles were automatically separated in order to eliminate the effect of individual differences in duration. Furthermore, an equal number of samples were collected for each class to improve the balance and representativeness of the dataset. Despite the fact that single-channel systems offer less data for real-time auscultation, a single-channel approach was adopted in this study to circumvent the complications associated with multichannel systems, especially in patients with physical difficulties. In this respect, various time-frequency representations, including spectrograms, scalograms, Mel-spectrograms and gammatonegrams, were used in combination with deep learning (DL) architectures such as Densenet201, VGG16, VGG19, InceptionV3, MobileNetV2 and ResNet50V2 to further improve the classification accuracy. Furthermore, a comparison was made between the performances of CNN, LSTM and SVM methods in the classification process. Unlike many recent high-accuracy lung sound classification studies based on fixed-duration segmentation or manually labelled respiratory cycles, this study presents a fully automatic respiratory cycle detection framework independent of individual breath duration. Additionally, class imbalance, one of the major limitations of existing datasets, is explicitly addressed by creating a balanced dataset containing an equal number of respiratory cycles per sound type. Furthermore, this study provides a systematic evaluation of biologically inspired time-frequency representations compared with different classification architectures. These methodological contributions distinguish the proposed approach from existing high-accuracy models and demonstrate that robust performance can be achieved using single-channel recordings under standardised conditions. Consequently, despite the limited data provided by the single-channel data collection method, the highest classification accuracy of 97.3% ± 1.9 was achieved by applying the gammatonegram representation via CNN with the Densenet201 architecture among the detailed signal processing and classification methods.

## 2. Material and Methods

**Figure 1:**
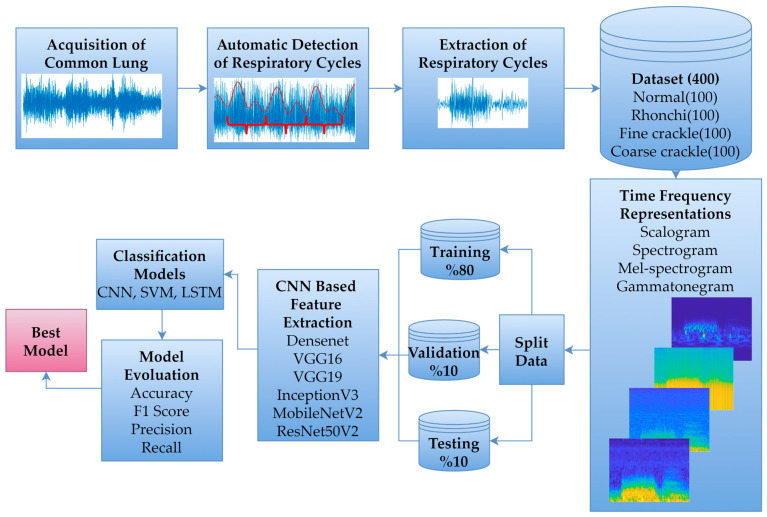
Proposed workflow diagram.

The primary framework of this study involved collaboration between two physicians with expertise in the field, who recorded and labelled common LSs across various respiratory cycles in accordance with standard auscultation procedures. Subsequently, a database was constructed by automatically identifying respiratory cycles from the recorded LSs. DL-based classification was then performed by applying different time-frequency representations to the data within this database. The proposed workflow is illustrated in Figure 1.

### 2.1. Data Acquisition

**Figure 2:**
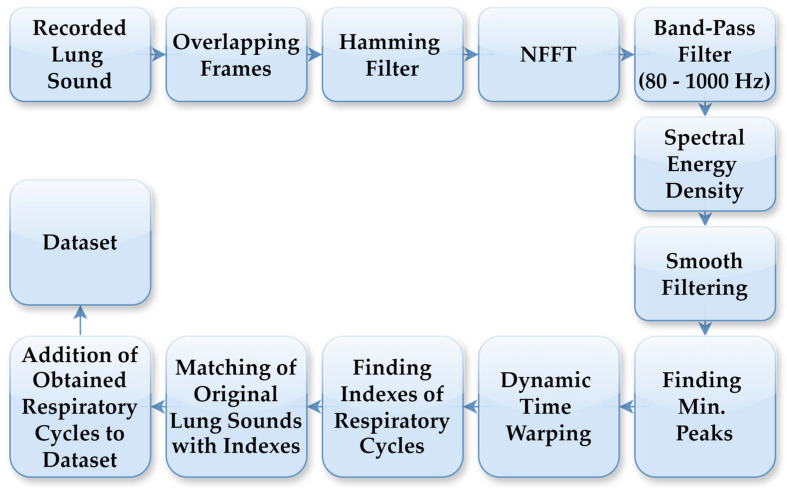
Process steps for automatic detection of respiratory cycles.

The LS data used in this study were collected from 94 individuals who visited the Department of Chest Diseases at Karadeniz Technical University. Data acquisition was conducted by expert physicians using a single-channel electronic stethoscope (Thinklabs ds32a+), operating within a frequency range of 20–2000 Hz. The recordings comprised respiratory cycles, characterised by sequential inhalation and exhalation of air through the respiratory system. Lung sound recordings were acquired at a sampling rate of 44.1 kHz. To accurately detect respiratory cycles with distinct characteristics for each individual, an automated segmentation system was employed independent of the cycle duration. In this segmentation process, FT was used to generate spectrograms from the LS recordings. Figure 2 illustrates the flowchart of the automatic respiratory cycle recognition process. As outlined in the flowchart, the LS recordings were initially divided into 10 ms frames, which were then processed using a Hamming filter. Next, an N-point Fast Fourier Transform (NFFT) was applied to all sub-frames using Hamming windows, and the spectrogram was computed with overlapping frames. Spectral energy was calculated within the 80–1000 Hz frequency band to suppress low-frequency motion artifacts and high-frequency noise. The lower cutoff frequency of 80 Hz was selected to suppress motion noise and heart sounds, while preserving the dominant spectral components of adventitious lung sounds, including crackles and rhonchi, consistent with prior lung sound analysis studies. The resulting energy signal was smoothed using a robust smoothing function to obtain repetitive respiratory patterns. Respiratory cycle boundaries were determined based on the similarity of these patterns using dynamic time warping (DTW). DTW comparisons employed a Euclidean distance measure, with amplitude normalisation applied to each pattern to reduce sensitivity to amplitude variations. Respiratory cycle durations were constrained between 1.25 s and 5.5 s, corresponding to the shortest and longest physiologically plausible breathing cycles observed in the dataset, respectively. Segments outside this range were treated as outliers and excluded from further analysis. The determined boundary points were applied on the original LSs to identify the respiratory cycles. This automated process was applied to LSs recordings containing between 3 and 7 respiratory cycles. The automatic segmentation outputs have been verified by physicians in a manner consistent with the previously defined method, and detailed threshold values, boundary determination criteria and error analyses have been comprehensively presented in our previous study [27].

**Figure 3:**
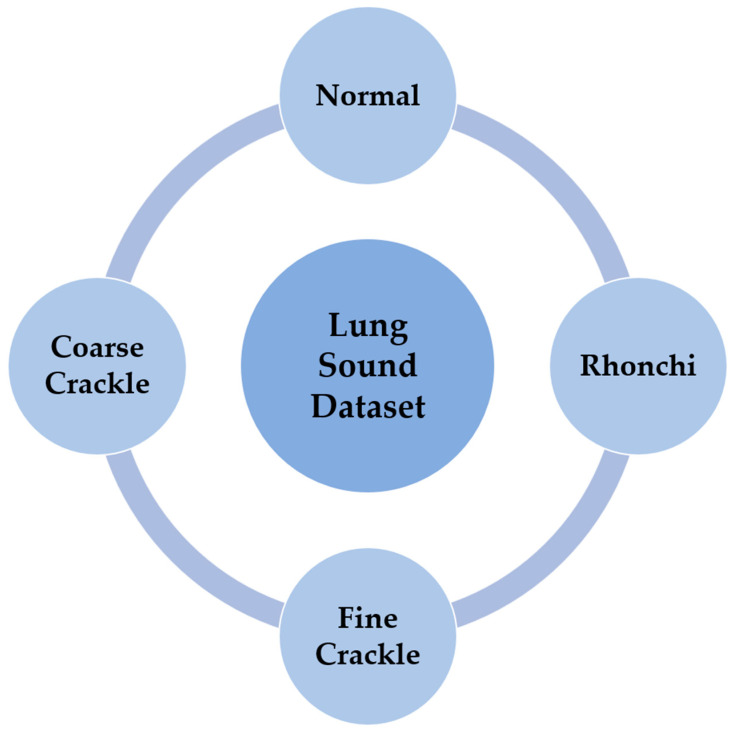
Common LSs in the dataset.

The automated segmentation process effectively identified respiratory cycles, enabling the precise extraction of various LSs. This study focused on normal, rhonchi, fine crackle and coarse crackle sounds, as shown in Figure 3, while wheezing was excluded due to its occurrence in healthy individuals during forced expiration and its audibility without a stethoscope due to its intensity [19]. Accordingly, a dataset comprising 100 respiratory cycles for each sound type was compiled, with the total number of cycles categorised into 100 normal and 300 abnormal respiratory cycles, as outlined in Table 1.

The study population represents a typical adult clinical cohort encountered in routine lung auscultation. It included adults aged 18–70 years, with a mean age of 45 ± 14 years, who had a clinical diagnosis and adequate recording quality. Records were taken in a quiet clinical environment, with individuals seated and breathing normally; individuals with serious comorbidities that could directly affect lung sounds were excluded from the study. To ensure labelling consistency, respiratory cycles from each subject were assigned to a single sound class.

### 2.2. Time-Frequency Representations

**Figure 4:**
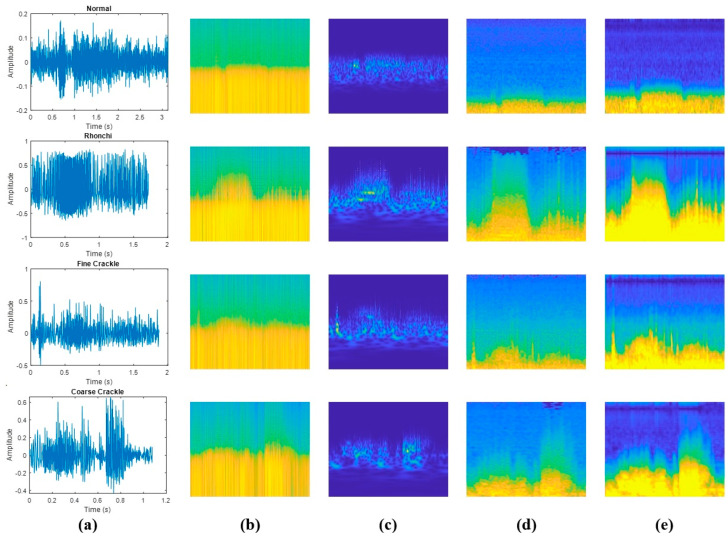
Comparison of various time−frequency representations of LSs types: (**a**) amplitude vs. time plots; (**b**) spectrograms; (**c**) scalograms; (**d**) Mel-spectrograms; (**e**) gammatonegrams.

Due to the advantages of two-dimensional spectral representations over one-dimensional time series data, this study comparatively analyses the performance of CNN-based methods for classifying LSs, which are particularly useful for image-based data and automatic feature extraction. Time-frequency analysis is widely used to characterise non-stationary signals in both the time and frequency domains, providing critical information about their temporal and spectral properties [28,29]. Thanks to these advantages, FT-, STFT- and WT-based time-frequency representations have been very important in extracting spectral features from respiratory sounds [7]. This approach is particularly useful for analysing respiratory sounds, which often exhibit complex patterns due to their time-dependent variations [5]. In this study, spectrogram, scalogram, Mel-spectrogram and gammatonegram techniques were applied to classify LSs. The use of time-frequency methods for different LS categories in the database is shown in Figure 4.

As shown in Figure 4, respiratory signals exhibit varying time durations due to differences in individual breathing patterns. However, since all signals are converted into fixed-size time-frequency representations prior to model input, this variability does not affect classification results. Additionally, these fixed−size representations ensure consistent input dimensions for the deep learning model and enable a fair comparison between signals of varying lengths.

#### 2.2.1. Scalogram

Scalograms show spectral content of audio signals over time and frequency, allowing us to study the changing frequency components. CWT decomposes the signal into its frequency components in different scales and it allows for a global view of frequency content across time. This multiscale strategy helps to identify specific patterns and anomalies in LSs analysis that are indicative of respiratory conditions and sends vital information with accurate time-frequency localisation. Scalograms were obtained using the continuous wavelet transform (CWT) implemented in MATLAB R2023b with the analytic “bump” mother wavelet. All signals were processed at a sampling rate of 44.1 kHz, and the default scale–frequency grid provided by MATLAB was used. The resulting time-frequency representations were exported at 300 dpi for CNN-based classification.

#### 2.2.2. Spectrogram

Spectrograms are the workhorse for visualising the frequency content of a signal and represent energy in the spectrum. This approach involves analysis of the frequency content of a signal by windowing the same using STFT. In this approach, the signal is partitioned into intervals of time, upon which an FT is performed. In the application of biomedicine signals (e.g., LS), this approach enables the detection of the typical frequency rhythms as well as temporal changes. Spectrograms were computed using the short-time Fourier transform (STFT) from signals sampled at 44.1 kHz. A frame length of 128 samples with a 50% overlap (64 samples) was used, and the FFT length was set to 1024. Power spectral density values were displayed using logarithmic magnitude scaling (10·log10(P)), and the frequency axis was shown on a logarithmic scale. Resulting spectrogram images were exported at 300 dpi for subsequent analysis.

#### 2.2.3. Mel-Spectrogram

The Mel-spectrogram is a solution for audio frequency analysis by accounting for the Mel scale as well, which closely resembles human perception of sound. The psychoacoustic design of this scale may increase frequency representation resolution specifically in medical applications, for example LS analysis. The approach adds the analysis ability of traditional spectrograms by using a Mel frequency filter bank [30]. Mel-spectrograms were generated from recordings sampled at 44.1 kHz using a periodic Hann window of 2048 samples with an overlap length of 2024 samples (hop size: 24 samples). The FFT length was set to 4096. A total of 128 Mel filter bands were used, covering the frequency range from 62.5 Hz to 15 kHz. The frequency axis was displayed on a logarithmic scale, and Mel-spectrogram images were exported at 300 dpi.

#### 2.2.4. Gammatonegram

Gammatonegram is a time-frequency representation based on Gammatone filter bank, which derives from the human auditory model as one of biologically-motivated filtering. This technique, which is suitable for analysing complex signals of short duration like lung sounds, has a better frequency resolution and less noise sensitivity than other time-frequency transforms [31]. Gammatonegram features were computed from signals sampled at 44.1 kHz using a 64-channel gammatone filter bank. Time-frequency magnitudes were integrated using a 25 ms analysis window (TWIN = 0.025 s) with a 10 ms hop size (THOP = 0.010 s). The filter bank was configured with a minimum center frequency of 50 Hz and ERB-spaced center frequencies extending up to the Nyquist limit (sr/2), using the time-domain ERB filter bank implementation (USEFFT = 0; WIDTH = 1.0). The resulting gammatonegram magnitudes were converted to log scale using 20·log10(·). For visualization in Figure 4, the displayed dynamic range was limited to [−90, −30] dB; exported images were saved at 300 dpi.

Theoretically, spectrogram and Mel-spectrogram representations are computationally lighter than scalogram and gammatonegram approaches, with scalograms being the most demanding due to multiscale analysis. All time-frequency representations were resized to a fixed spatial resolution of 256 × 256 pixels with three channels prior to being passed to the convolutional neural networks. This input size was selected to ensure compatibility with ImageNet-pretrained CNN backbones used in this study.

### 2.3. Convolutional Neural Networks

**Figure 5:**
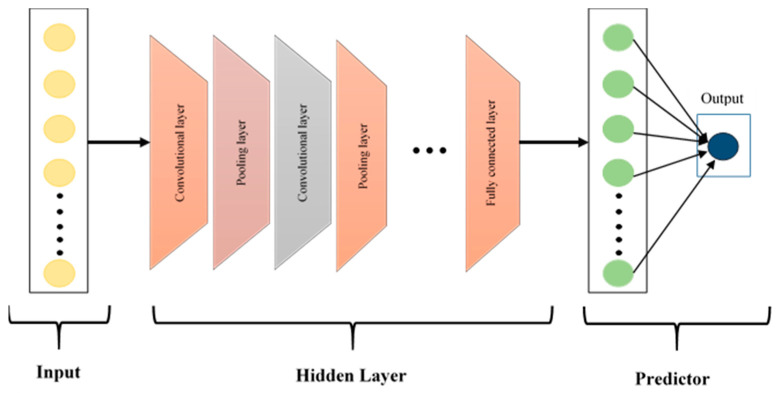
The architecture of the CNN model.

CNNs, first introduced by LeCun et al. [32], have been widely used in the classification of biomedical signals because they automatically find useful features from complex data. The CNN architecture shown in Figure 5 consists of three basic layers: convolutional, pooling and fully connected layers. Convolutional layers are used to create feature maps by extracting local patterns through the application of filters to the input data. Pooling layers then reduce dimensionality while preserving essential information, which enhances performance and lowers computational demands. Finally, fully connected layers aggregate these learned features for classification, contributing to high diagnostic accuracy in various medical tasks.

**Figure 6:**
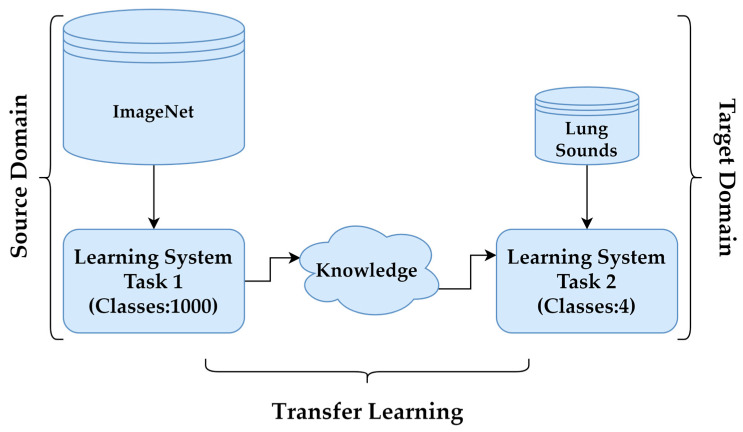
Transfer learning.

In cases where obtaining labelled data, such as LSs, is challenging, TL provides an effective solution to improve model performance. By leveraging pre-trained models, TL reduces the need for extensive training data while enhancing accuracy and efficiency. Pre-trained models, initially developed for tasks like object recognition on large datasets such as ImageNet, serve as feature extractors in this process. In this study, the DenseNet201, InceptionV3, MobileNetV2, ResNet50V2, VGG16 and VGG19 architectures were employed for feature extraction, with adaptations made via TL to suit the specific target task. These CNN architectures, along with their key features, are summarised in Table 2. The process, illustrated in Figure 6, involved transforming the baseline model by adding a custom output layer tailored to the classification of LSs.

### 2.4. Proposed Model

Given that the duration of respiratory cycles in different LSs varies depending on the patient, time-frequency representations were utilised to standardise the data to a uniform size in the study. These representations were subsequently adjusted to a suitable input size (256 × 256 pixels) to match the default input requirements of the classification models, ensuring a fair comparison across architectures. Additionally, to maintain the evaluation consistency of the classification models and ensure a clear separation between training, validation and testing sets, the dataset was split into three sections: 80% for training, 10% for validation and 10% for testing. Data splitting was performed at the respiratory cycle level rather than at the subject level, meaning that individual respiratory cycles were randomly assigned to training, validation and test sets. To reduce the effect of randomness introduced by the data splitting process and to obtain a more reliable performance estimate, the classification experiments were repeated ten times using different random partitions of the dataset. For each experimental configuration, the overall classification performance was reported in terms of mean accuracy and standard deviation across these repetitions, providing insights into both model performance and stability. Furthermore, class-wise precision, recall and F1 scores were computed for a representative run whose overall accuracy was closest to the mean value obtained over multiple repetitions. During CNN-based model training, the number of epochs was set to 100, and early stopping with a patience of 15 epochs was applied to mitigate overfitting. A batch size of 16 was utilised, and the discrepancy between predicted and true labels was minimised using the categorical cross-entropy loss function. The Adam optimiser was employed to further reduce this loss. In addition to the method used on CNN classification, a hybrid method on CNN-SVM and CNN−LSTM was also used in order to investigate the classifier dependency of feature extractor.

**Figure 7:**
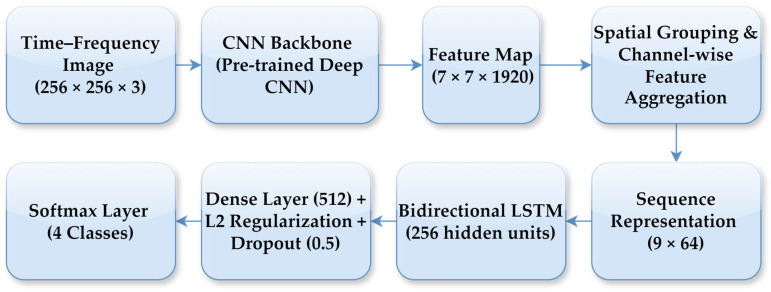
Block diagram of the proposed CNN–LSTM architecture.

In the CNN–LSTM model, a high-level feature map of size 7 × 7 × 1920 was extracted from the final convolutional block of the CNN backbone for each time-frequency representation. To enable sequential modelling, this feature map was transformed into a compact sequence representation through spatial grouping and channel-wise feature aggregation. Specifically, the spatial feature map was partitioned into a fixed number of regions, and aggregation was applied within each region to obtain low-dimensional feature vectors, resulting in a sequence representation with 9-time steps and 64 features per step. This feature reshaping step enables the LSTM to operate on a compact sequence representation while preserving local time-frequency structure. As part of the fine-tuning strategy, only a limited number of top layers of the CNN backbone were set as trainable, while earlier layers retained their ImageNet-pretrained weights, allowing the model to adapt high-level representations without overfitting given the limited dataset size. A bidirectional LSTM with 256 hidden units was employed to learn temporal dependencies in both forward and backward directions across the feature sequence. The LSTM output was followed by a fully connected layer with 512 neurons, where L2 regularisation and a dropout rate of 0.5 were applied to mitigate overfitting. Finally, a dense layer with a softmax activation function produced the probabilities for the four lung sound classes. The model was trained using the Adam optimiser with an initial learning rate of 0.001, which was dynamically adjusted based on the validation loss. The overall CNN–LSTM architecture and the feature reshaping process are illustrated in Figure 7.

The CNN–SVM configuration was included to explicitly decouple deep feature extraction from the classification stage and to assess the classifier dependency of the learned representations. In this approach, the pretrained CNN backbone was used solely as a fixed feature extractor, resulting in zero trainable parameters on the CNN side. A linear SVM was preferred due to the high dimensionality and strong linear separability of CNN-derived features, as well as its robustness under limited data conditions. This configuration provides a lightweight and stable baseline, enabling a direct comparison between end-to-end deep learning and hybrid learning strategies in terms of performance and overfitting behaviour. In the CNN configuration, the convolutional layers of the pretrained backbone were entirely frozen and used strictly as fixed feature extractors, with only the newly added classification layers trained. In this configuration, no additional dropout or L2 regularisation was applied within the CNN backbone, and overfitting control relied primarily on transfer learning and early stopping. In contrast, the CNN–LSTM configuration employed partial fine-tuning to allow limited adaptation of high-level features. Specifically, only the last 100 layers of the DenseNet201 backbone were set as trainable, while earlier layers retained their ImageNet-pretrained weights. This choice reflects a balance between adapting high-level semantic representations to lung sound characteristics and limiting the number of trainable parameters under limited data conditions.

## 3. Results

The most prevalent approach to assessing the efficacy of a classification model is through the utilisation of the accuracy metric, which is determined by the ratio of accurate predictions to the total number of predictions (Equation (1)). The accuracy metric offers an effective means of comparison, particularly when the number of instances of each class is equal. In addition, there are four cases when the prediction results of the model are compared with the actual values: positive predictions for positively labelled data (TP), negative predictions for positively labelled data (FN), negative predictions for negatively labelled data (TN) and positive predictions for negatively labelled data (FN). The numbers belonging to these values are used in the calculation of precision (Equation (2)), recall (Equation (3)) and F1 score (Equation (4)) comparison metrics.

(1) $$Accuracy=\frac{TP+TN}{TP+FP+TN+FN}$$

(2) $$Precision\;(Pre.)=\frac{TP}{TP+FP}$$

(3) $$Recall\;(Rec.)=\frac{TP}{TP+FN}$$

(4) $$F1\;score\;(F1)=2\times \frac{Precision\times Recall}{Precision+Recall}$$

The classification performance of different CNN architectures and time-frequency representations used for deep learning-based lung sound classification is reported in Table 3. The accuracy values are presented as mean ± standard deviation of the test accuracy across ten independent experimental runs.

**Figure 8:**
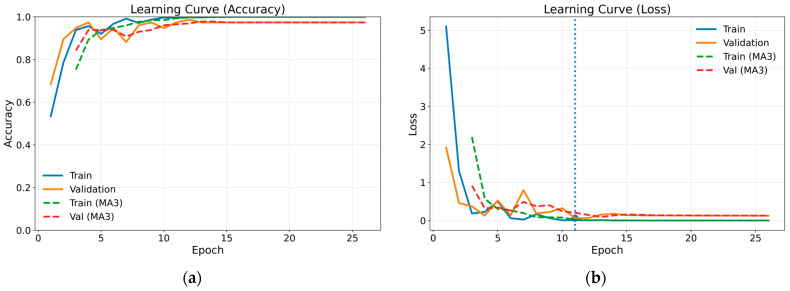
Training and validation learning curves in terms of loss and accuracy for the proposed model: (**a**) accuracy; (**b**) loss.

The confusion matrix for the method combining gammatonegram time-frequency representation and DenseNet 201 architecture, which proved the most successful of the methods employed, is presented in Table 4. The confusion matrix provides comprehensive insights into the efficacy of classification by presenting the actual and predicted values in a single matrix. In the case of a classification model that is operating at optimal performance, all data values are represented on the leading diagonal of the confusion matrix. The off-diagonal values represent instances of misclassification. Figure 8 illustrates the training and validation learning curves in terms of loss and accuracy for the most successful method. Both loss and accuracy curves exhibit stable convergence, with close alignment between training and validation performance, indicating effective regularisation and the absence of severe overfitting. The marked point on the loss curve corresponds to the epoch at which the validation loss reaches its minimum, representing the optimal model state selected by the early stopping criterion. To enhance visual clarity and suppress short-term fluctuations arising from random data splits, a moving average with a window size of three epochs (MA3) was applied and is shown using dashed curves. For class-wise performance analysis, a representative test run whose overall accuracy was closest to the mean value obtained across repeated experiments was selected, and the corresponding class-wise precision, recall and F1 scores are reported in Table 5.

Table 6 presents the performance of the gammatonegram-based DenseNet201 model under different data splitting ratios, reported as mean ± standard deviation over ten repeated experiments.

## 4. Conclusions and Discussion

This study assessed the efficacy of deep learning-based architectures in classifying common LSs through the utilisation of disparate time-frequency representations. The experimental results offer guidance on the efficacy of each approach and representation in accurately classifying fine crackles, coarse crackles, rhonchi and normal LSs. It was demonstrated that the method yielded more stable results when automatic detection of the respiratory cycles was employed. The DenseNet201-based CNN classifier architecture shows the highest performance with a 97.3% ± 1.9 accuracy, especially for lung sounds represented by gammatonegrams. This outcome is attributed to the capacity of gammatonegram to discern fine spectral nuances in lung sounds with greater efficacy, along with its resilience to noise components. Additionally, the DenseNet201 architecture’s aptitude for enhancing feature transfer and stability during the learning process, facilitated by dense connections, contributes to the effectiveness of the system. Specifically, the confusion matrix of the most successful method in Table 4 demonstrates that the normal class is classified with a 100% accuracy, while the other classes are classified with high accuracy, indicating that the model can clearly distinguish between classes. Table 5 also shows the strengths and weaknesses of the methods used on CNN-based classification, which is the most successful method in Table 5, in terms of class-based precision, recall and F1 scores in terms of feature extraction and time-frequency representations. The combination of gammatonegram with DenseNet201, in particular, represents an effective approach with the potential to facilitate the development of enhanced diagnostic tools and more precise methods of lung condition detection. As shown in Table 6, the gammatonegram–DenseNet201 model achieves its most stable and highest performance under the 80/10/10 data splitting ratio. Table 7 provides an overview of selected DL-based studies from the literature and a comparative analysis of the present study. One of the fundamental limitations of LS studies is the absence of a standard methodology for audio recording and pre-processing. This situation may lead to inconsistencies in the classification of sounds. Furthermore, the performance metric used in the ICBHI 2017 database is the challenge score, which combines sensitivity and specificity with specific weights, and differs from the accuracy metric commonly reported in the literature. Therefore, the challenge score values of some studies in Table 6 cannot be directly compared with the accuracy values of the proposed method. Despite all these limitations, when compared to other studies, the proposed method demonstrates high performance and stability in classification. Thanks to the automatic separation of respiratory cycles and appropriate time-frequency representations, it has been shown that single-channel LSs are as successful as multi-channel LSs in DL-based classification.

The automatic respiratory cycle detection and cycle-based classification approach presented in this study offers a structure that directly corresponds to the auscultation process used in clinical practice. The confusion matrix of the best-performing gammatonegram–DenseNet201 model further illustrates its clinical relevance. In particular, the high recall values observed for abnormal lung sound classes indicate strong sensitivity to pathological respiratory events, while the correct identification of normal cycles reflects high specificity. Misclassifications mainly occur between acoustically similar classes, such as fine and coarse crackles, which is consistent with clinical observations and reflects the inherent ambiguity of these sound patterns. Combining predictions obtained from multiple respiratory cycles has the potential to facilitate the actual patient assessment process. The proposed method can be integrated with digital stethoscopes, portable recording devices or mobile health applications and used as a decision support tool in primary care screening, outpatient follow-up and remote healthcare services. The absence of additional manual processing requirements, thanks to automatic cycle detection, increases the method’s applicability in busy clinical settings. However, multicentre validation studies in different patient groups and recording conditions will further strengthen the method’s clinical generalisability. Therefore, designing a device that performs fully automatic LS classification is our primary goal in future studies.

## Figures and Tables

**Table 1:** Description of the database content.

Sound Type	Number of Subjects	Number of Respiratory Cycles
Normal (N)	30	100
Rhonchi (R)	23	100
Fine Crackle (FC)	20	100
Coarse Crackle (CC)	21	100
Total Database	94	400

**Table 2:** Key features of the CNN architectures used.

Architecture	Key Features
DenseNet201	Densely connected layers, improved information flow, reduces overfitting and fewer parameters for DNN.
VGGNet	Deep architecture (16 or 19 layers), 3 × 3 filters, ReLU activation and pooling layers for feature reduction.
InceptionV3	Modular structure, multiscale feature learning and smaller filters (1 × 1) for computational efficiency.
MobileNetV2	Inverted residual blocks, depthwise convolutions, lightweightness and efficiency for mobile applications.
ResNet50V2	Residual learning, 50 layers, block-wise connections and enhanced performance in deeper networks.

**Table 3:** Test classification accuracies of the methods.

Time-Frequency Representation	Feature Extractor	CNN Classifier Accuracy %	LSTM Classifier Accuracy %	SVM Classifier Accuracy %
Spectrogram	DenseNet 201	85.3 ± 2.3	80.0 ± 2.8	75.0 ± 3.1
	VGG16	75.0 ± 3.4	70.0 ± 3.8	67.5 ± 4.0
	VGG19	82.5 ± 2.9	77.5 ± 3.2	72.5 ± 3.6
	InceptionV3	77.5 ± 3.6	77.5 ± 3.4	72.5 ± 3.9
	MobileNetV2	82.5 ± 2.7	80.0 ± 3.0	77.5 ± 3.3
	ResNet50V2	75.0 ± 3.5	70.0 ± 3.9	72.5 ± 3.7
Mel-spectrogram	DenseNet 201	90.3 ± 2.1	85.0 ± 2.6	82.5 ± 2.9
	VGG16	82.5 ± 3.0	75.0 ± 3.4	70.0 ± 3.8
	VGG19	87.5 ± 2.5	80.0 ± 3.0	75.0 ± 3.3
	InceptionV3	75.0 ± 3.7	75.0 ± 3.6	72.5 ± 3.9
	MobileNetV2	80.0 ± 3.2	72.5 ± 3.6	75.0 ± 3.5
	ResNet50V2	80.0 ± 2.9	77.5 ± 3.3	82.5 ± 2.8
Scalogram	DenseNet 201	92.5 ± 2.0	75.0 ± 3.5	82.5 ± 3.0
	VGG16	90.0 ± 2.6	75.0 ± 3.7	67.5 ± 4.2
	VGG19	87.5 ± 2.8	80.0 ± 3.2	67.5 ± 4.0
	InceptionV3	80.0 ± 3.6	85.0 ± 3.1	60.0 ± 4.5
	MobileNetV2	85.0 ± 3.0	85.0 ± 2.9	77.5 ± 3.4
	ResNet50V2	87.5 ± 2.7	85.0 ± 3.0	80.0 ± 3.3
Gammatonegram	**DenseNet 201**	**97.3 ± 1.9**	82.5 ± 3.0	85.0 ± 2.8
	VGG16	87.5 ± 2.6	85.0 ± 2.9	77.5 ± 3.4
	VGG19	85.0 ± 3.0	82.5 ± 3.2	80.0 ± 3.3
	InceptionV3	80.0 ± 3.6	77.5 ± 3.8	72.5 ± 4.0
	MobileNetV2	87.5 ± 2.7	82.5 ± 3.1	77.5 ± 3.5
	ResNet50V2	85.0 ± 2.8	75.0 ± 3.9	72.5 ± 4.1

**Table 4:** Confusion matrix of the most successful method.

	Normal	Rhonchi	Fine Crackle	Coarse Crackle
Normal	10	0	0	0
Rhonchi	1	9	0	0
Fine Crackle	0	0	10	0
Coarse Crackle	0	0	0	10

**Table 5:** Class-wise performance comparison of different classification methods using precision, recall and F1 score.

		Spectrogram			Mel-Spectrogram			Scalogram			Gammatonegram		
Model	Class	Pre.	Rec.	F1	Pre.	Rec.	F1	Pre.	Rec.	F1	Pre.	Rec.	F1
DenseNet201	FC	0.75	0.90	0.82	0.82	0.90	0.86	0.83	1.00	0.91	0.91	1.00	0.95
	CC	0.88	0.70	0.78	1.00	0.80	0.89	1.00	0.90	0.95	1.00	0.90	0.95
	R	0.82	0.90	0.86	0.82	0.90	0.86	0.89	0.80	0.84	1.00	1.00	1.00
	N	1.00	0.90	0.95	1.00	1.00	1.00	1.00	1.00	1.00	1.00	1.00	1.00
VGG16	FC	0.89	0.80	0.84	0.88	0.70	0.78	0.83	1.00	0.91	0.90	0.90	0.90
	CC	0.58	0.70	0.64	0.71	1.00	0.83	0.90	0.90	0.90	0.86	0.60	0.71
	R	0.56	0.50	0.53	0.88	0.70	0.78	0.89	0.80	0.84	0.77	1.00	0.87
	N	1.00	1.00	1.00	0.90	0.90	0.90	1.00	0.90	0.95	1.00	1.00	1.00
VGG19	FC	0.78	0.70	0.74	1.00	0.80	0.89	0.75	0.90	0.82	0.80	0.80	0.80
	CC	1.00	0.80	0.89	0.75	0.90	0.82	0.90	0.90	0.90	0.78	0.70	0.74
	R	0.67	0.80	0.73	0.80	0.80	0.80	0.89	0.80	0.84	0.82	0.90	0.86
	N	0.91	1.00	0.95	1.00	1.00	1.00	1.00	0.90	0.95	1.00	1.00	1.00
InceptionV3	FC	0.80	0.80	0.80	0.78	0.70	0.74	0.67	0.80	0.73	0.73	0.80	0.76
	CC	0.64	0.70	0.67	0.88	0.70	0.78	0.89	0.80	0.84	0.75	0.60	0.67
	R	0.70	0.70	0.70	0.55	0.60	0.57	0.78	0.70	0.74	0.75	0.90	0.82
	N	1.00	0.90	0.95	0.83	1.00	0.91	0.90	0.90	0.90	1.00	0.90	0.95
MobileNetV2	FC	0.75	0.90	0.82	0.78	0.70	0.74	0.73	0.80	0.76	0.83	1.00	0.91
	CC	0.86	0.60	0.71	0.73	0.80	0.76	0.89	0.80	0.84	1.00	0.60	0.75
	R	0.80	0.80	0.80	0.73	0.80	0.76	0.82	0.90	0.86	0.75	0.90	0.82
	N	0.91	1.00	0.95	1.00	0.90	0.95	1.00	0.90	0.95	1.00	1.00	1.00
ResNet50V2	FC	0.64	0.90	0.75	0.69	0.90	0.78	0.80	0.80	0.80	0.82	0.90	0.86
	CC	1.00	0.50	0.67	0.78	0.70	0.74	0.82	0.90	0.86	0.80	0.80	0.80
	R	0.64	0.70	0.67	0.86	0.60	0.71	0.89	0.80	0.84	0.78	0.70	0.74
	N	0.90	0.90	0.90	0.91	1.00	0.95	1.00	1.00	1.00	1.00	1.00	1.00

**Table 6:** Test accuracy results obtained under different data splitting ratios for the best-performing classifier.

Data Splitting Rate (Train/Validation/Test)	Accuracy % (Mean ± Standard Deviation)
80/10/10	97.3 ± 1.9
70/10/20	95.6 ± 2.4
60/10/30	93.8 ± 2.9
50/10/40	91.5 ± 3.6

**Table 7:** Comparison of DL-based studies on the classification of LSs.

References	Data Size and Type (Multi/Single Ch.)	Dataset Creation Methods	Classification Methods	Results
[33]	171 normal, 33 wheeze, 19 crackle, 4 wheeze and crackle and single-channel	Four-second recordings	Spectrograms with a semi-supervised DL method	AUC; 86% wheeze; 74% crackle.
[9]	886 wheezes, 1864 crackles 506 wheezes+ crackles and 3642 normal (ICBHI 2017)	Manually labelled respiratory cycle durations	Mel-spectrograms with a CNN-RNN based model	Score: 71.81%
[5]	297 crackles, 298 wheezes, 101 rhonchi and single-channel	Divided LS into 6 s each with a 50% overlapping	Mel-spectrograms with VGG16	Accuracy: 85.7%
[15]	702 normal, 436 crackles and 295 wheezes	Manually labelled respiratory cycle durations	Gammatonegrams With ResNet-50	Accuracy: 98.80%
[17]	886 wheezes, 1864 crackles, 506 wheezes+ crackles and 3642 normal (ICBHI 2017)	Cycles with a duration that exceeds 2.7 s were cropped, preserving the first 2.7 s, while cycles with a duration lower than 2.7 s were expanded using sample padding.	Spectrograms with CNN−LSTM and loss function selection	Accuracy: 76.39%
[21]	105 normal, 116 crackle and 73 rhonchi	Fixed 15 s period	Spectrograms with VGG16-CNN-SVM	Accuracy: 83%
[22]	886 wheezes, 1864 crackles, 506 wheezes + crackles and 3642 normal (ICBHI 2017)	Zero padding for a constant duration of 6 s	Scalograms with a Parallel Convolutional Autoencoder (CAE), LSTM	Accuracy: 80%
[24]	886 wheezes, 1864 crackles, 506 wheezes + crackles and 3642 normal (ICBHI 2017)	Sampling time: 6 s	Mel-spectrograms with VGG19 and the Convolutional Block Attention Module (CBAM)	Score: 75.73%
**Proposed**	100 normal, 100 rhonchi, 100 fine crackles, 100 coarse crackles and single-channel	Automatic	Gammatonegrams with DenseNet201	Accuracy: 97.0%

## Data Availability

The data that support the findings of this study are not openly available due to reasons of sensitivity and are available from the corresponding author upon reasonable request.
